# Family Caregivers and Support: Implications for Work Strain and Formal Service Use

**DOI:** 10.1093/geroni/igab046.2966

**Published:** 2021-12-17

**Authors:** Joseph Svec, Jeongeun Lee

**Affiliations:** Iowa State University, Ames, Iowa, United States

## Abstract

In the US, many employed caregivers make professional adjustments, exacerbating already tenuous balances between work and life. Using the framework of the Stress Process Model (SPM), current research examines the sources of support (both formal and informal) and the contextual factors that facilitate or impede caregiver support. In this research, we examine whether and to what extent caregiver work strain is ameliorated by the presence of additional family caregivers and formal service use. This study utilizes data provided by the National Study of Caregiving (NSOC) data. Using panel methods for the pooled waves, we analyze the associations between work-strain and the number of additional caregivers with utilization of formal support (such as paid service support). Preliminary analyses align with the Stress Process Model as additional caregivers for each respective care-recipient is associated with lower levels of work strain. On the other hand, utilization of formal services (paid help and Medicaid funding) is positively associated with work strain. These findings suggest that the number of additional caregivers can reduce the negative impact of caregiving on work related strain among employed caregivers. That is, multiple caregivers may be more reflective of cooperative arrangements which offset work disruptions that occur with the onset of caregiving. In addition, formal sources may more frequently be used as a last resort to address caregiver burnout. Ongoing analyses are examining changes in the number of caregivers and its impact on disruptive work event, which could lead to financial outcomes for caregivers.

